# Supplementary material for: Melioidosis Queensland: An analysis of clinical outcomes and genomic factors
Source: PLoS Negl Trop Dis. 2023 Oct 12;17(10):e0011697. doi: 10.1371/journal.pntd.0011697 (PMC10610085; doi:10.1371/journal.pntd.0011697)
Supplement: S1 Table — (DOCX) [file pntd.0011697.s001.docx]

**S1 Table. Reference strains used to create custom virulence factor database**

| **Virulence factors** | **Reference strain** | **Locus tag/accession** |
| --- | --- | --- |
| YLF | *B. pseudomallei* K96243 chromosome 2 | BPSS0120 |
| YLF | *B. pseudomallei* K96243 chromosome 2 | BPSS0123 |
| YLF | *B. pseudomallei* K96243 chromosome 2 | BPSS0122 |
| YLF | *B. pseudomallei* K96243 chromosome 2 | BPSS0121 |
| LPS_B | *B. pseudomallei* 579 | NZ_ACCE01000003 |
| LPS_A | *B. pseudomallei* K96243 chromosome 1 | NC_006350 |
| LPS_B2 | *B. pseudomallei* MSHR840 | GU574442 |
| bimA_Bm_ | *B. pseudomallei* 668 | BURPS668_A2118 |
| BTFC cluster | *B. pseudomallei* 305 | EF377328.1 |
| fhaB3 | *B. pseudomallei* K96243 chromosome 2 | BPSS2053 |
