# Supplementary material for: Melioidosis Queensland: An analysis of clinical outcomes and genomic factors
Source: PLoS Negl Trop Dis. 2023 Oct 12;17(10):e0011697. doi: 10.1371/journal.pntd.0011697 (PMC10610085; doi:10.1371/journal.pntd.0011697)
Supplement: S2 Table — (DOCX) [file pntd.0011697.s002.docx]

**S2 Table. Metadata of the previously published genomes**

| **Acc ID** | **ST** | **Year** | **Country** | **Geographic Group** |
| --- | --- | --- | --- | --- |
| ERR539769 | 32 | 2007 | China | Asia |
| ERR539767 | 70 | 2007 | China | Asia |
| ERR539770 | 70 | 1987 | China | Asia |
| ERR539773 | 105 | 2008 | Cambodia | Asia |
| SRR1617372 | 129 | 1995 | Australia | Australasia |
| ERR178265 | 211 | 2010 | Vietnam | Asia |
| ERR539742 | 244 | 2002 | Australia | Australasia |
| ERR539752 | 245 | 2004 | Australia | Australasia |
| ERR539744 | 255 | 2002 | Australia | Australasia |
| ERR539758 | 266 | 2006 | Australia | Australasia |
| ERR539757 | 450 | 2005 | Australia | Australasia |
| ERR539759 | 514 | 2006 | Australia | Australasia |
| ERR539760 | 515 | 2006 | Papua New Guinea | Australasia |
| ERR539761 | 573 | 2007 | Australia | Australasia |
| ERR539750 | 797 | 2003 | Australia | Australasia |
| ERR539764 | 861 | 2009 | Australia | Australasia |
| ERR298347 | 82 | 1956 | Chad | Africa |
| ERR298756 | 6 | 1973 | Burkina_Faso | Africa |
| ERR298772 | 707 | 2010 | Nigeria | Africa |
| ERR298779 | 82 | 1956 | Chad | Africa |
| ERR311038 | unknown | 2004 | Mauritius | Africa |
| ERR403714 | 1128 | 2012 | Gabon | Africa |
| ERR403716 | 1127 | 2013 | Gabon | Africa |
| SRR3145394 | 1121 | unknown | Burkina Faso | Africa |
| SRR3145395 | 1122 | unknown | Burkina Faso | Africa |
| SRR3145396 | 1043 | unknown | Madagascar | Africa |
| ERR298357 | 92 | 2010 | Martinique | America |
| ERR298359 | 92 | 2010 | Martinique | America |
| ERR298753 | 92 | 2003 | Brazil | America |
| ERR298754 | 92 | 2006 | Brazil | America |
| ERR298778 | unknown | 2007 | Virgin_Islands | America |
| ERR311035 | 95 | 1982 | Puerto_Rico | America |
| ERR311039 | 92 | 1998 | Puerto_Rico | America |
| ERR351981 | 12 | 1976 | Venezuela | America |
| ERR351982 | 11 | 1962 | Ecuador | America |
| SRR7140691 | 0 | unknown | Mexico | America |
| ERR162597 | 162 | 2003 | Laos | Asia |
| ERR162598 | 511 | 2004 | Laos | Asia |
| ERR162599 | 495 | 2004 | Laos | Asia |
| ERR162600 | 489 | 2005 | Laos | Asia |
| ERR162601 | 504 | 2004 | Laos | Asia |
| ERR162602 | 205 | 2005 | Laos | Asia |
| ERR162603 | 500 | 2004 | Laos | Asia |
| ERR162604 | 513 | 2004 | Laos | Asia |
| ERR162605 | 491 | 2004 | Laos | Asia |
| ERR162606 | 488 | 2005 | Laos | Asia |
| ERR162607 | 507 | 2005 | Laos | Asia |
| ERR162608 | 512 | 2005 | Laos | Asia |
| ERR162609 | unknown | 2009 | Laos | Asia |
| ERR162610 | 16 | 2009 | Laos | Asia |
| ERR162611 | 47 | 2009 | Laos | Asia |
| ERR162612 | 70 | 2001 | Thailand | Asia |
| ERR162613 | 167 | 2001 | Thailand | Asia |
| ERR162614 | 54 | 2001 | Thailand | Asia |
| ERR162615 | 58 | 2001 | Thailand | Asia |
| ERR162617 | 60 | 2001 | Thailand | Asia |
| ERR162618 | 174 | 2001 | Thailand | Asia |
| ERR162619 | 48 | 2001 | Thailand | Asia |
| ERR162620 | 34 | 2001 | Thailand | Asia |
| ERR162621 | 33 | 2001 | Thailand | Asia |
| ERR162622 | 317 | 1995 | Thailand | Asia |
| ERR162623 | 309 | 1992 | Thailand | Asia |
| ERR162624 | 316 | 2002 | Thailand | Asia |
| ERR162625 | 229 | 2001 | Thailand | Asia |
| ERR162626 | 230 | 1998 | Thailand | Asia |
| ERR178245 | 694 | 2008 | Cambodia | Asia |
| ERR178250 | 693 | 2007 | Cambodia | Asia |
| ERR178251 | 690 | 2007 | Cambodia | Asia |
| ERR178253 | 494 | 2006 | Cambodia | Asia |
| ERR178254 | 510 | 2006 | Cambodia | Asia |
| ERR178256 | 67 | 2008 | Vietnam | Asia |
| ERR178258 | 163 | 2009 | Vietnam | Asia |
| ERR178259 | 871 | 2009 | Vietnam | Asia |
| ERR178260 | 67 | 2009 | Vietnam | Asia |
| ERR178261 | 884 | 2010 | Vietnam | Asia |
| ERR178262 | unknown | 2010 | Vietnam | Asia |
| ERR178263 | unknown | 2010 | Vietnam | Asia |
| ERR178264 | 653 | 2010 | Vietnam | Asia |
| ERR178266 | 916 | 2010 | Vietnam | Asia |
| ERR178267 | 67 | 2010 | Vietnam | Asia |
| ERR178268 | 367 | 2010 | Vietnam | Asia |
| ERR178269 | 653 | 2011 | Vietnam | Asia |
| ERR298348 | 169 | 1947 | Vietnam | Asia |
| ERR298349 | 56 | 1947 | Vietnam | Asia |
| ERR298350 | 56 | 1947 | Vietnam | Asia |
| ERR298351 | 288 | 1953 | Vietnam | Asia |
| ERR298352 | 169 | 1948 | Vietnam | Asia |
| ERR298353 | 51 | 1935 | Singapore | Asia |
| ERR298354 | 661 | 1967 | Sabah(Malaysia) | Asia |
| ERR298360 | 849 | 2010 | Cambodia | Asia |
| ERR298747 | 56 | 1998 | Bangladesh | Asia |
| ERR298748 | 43 | 1999 | Bangladesh | Asia |
| ERR298749 | 71 | 1999 | Bangladesh | Asia |
| ERR298750 | unknown | 2003 | Bangladesh | Asia |
| ERR298751 | 1007 | 2006 | Bangladesh | Asia |
| ERR298752 | unknown | 2010 | Bangladesh | Asia |
| ERR298755 | 169 | 2003 | Brunei | Asia |
| ERR298757 | unknown | 1996 | China | Asia |
| ERR298758 | 50 | 1996 | China | Asia |
| ERR298759 | 344 | 2005 | India | Asia |
| ERR298760 | unknown | 2010 | India | Asia |
| ERR298763 | 51 | 1977 | Malaysia | Asia |
| ERR298764 | 289 | unknown | Malaysia | Asia |
| ERR298765 | 46 | unknown | Malaysia | Asia |
| ERR298766 | 46 | unknown | Malaysia | Asia |
| ERR298767 | 438 | unknown | Malaysia | Asia |
| ERR298768 | 46 | unknown | Malaysia | Asia |
| ERR298769 | unknown | unknown | Malaysia | Asia |
| ERR298770 | 426 | 1999 | Malaysia | Asia |
| ERR298771 | 51 | 1999 | Malaysia | Asia |
| ERR311033 | 46 | 1960 | Bangladesh | Asia |
| ERR311037 | 43 | 1995 | India | Asia |
| ERR311047 | 232 | 1994 | Malaysia | Asia |
| ERR343795 | 218 | 2011 | Thailand | Asia |
| ERR343796 | 54 | 2011 | Thailand | Asia |
| ERR343806 | 206 | 2011 | Thailand | Asia |
| ERR343807 | 50 | 2011 | Thailand | Asia |
| ERR343810 | 50 | 2011 | Thailand | Asia |
| ERR343816 | 399 | 2011 | Thailand | Asia |
| ERR343837 | 9 | 2011 | Thailand | Asia |
| ERR539754 | 46 | 2005 | Indonesia | Asia |
| ERR539766 | 70 | 1987 | China | Asia |
| ERR539771 | 70 | 1999 | China | Asia |
| SRR12983327 | 1753 | 2018 | Myanmar | Asia |
| SRR12983328 | 1752 | 2018 | Myanmar | Asia |
| SRR12983329 | 346 | 2018 | Myanmar | Asia |
| SRR12983330 | 1371 | 2018 | Myanmar | Asia |
| SRR12983331 | 90 | 2018 | Myanmar | Asia |
| SRR12983332 | 56 | 2018 | Myanmar | Asia |
| SRR12983333 | 90 | 2018 | Myanmar | Asia |
| SRR12983334 | 56 | 2017 | Myanmar | Asia |
| SRR12983335 | 1766 | 2018 | Myanmar | Asia |
| SRR12983336 | 1765 | 2018 | Myanmar | Asia |
| SRR12983337 | 1770 | 2018 | Myanmar | Asia |
| SRR12983338 | 1753 | 2018 | Myanmar | Asia |
| SRR12983339 | 1753 | 2018 | Myanmar | Asia |
| SRR12983340 | 1753 | 2018 | Myanmar | Asia |
| SRR12983341 | 56 | 2017 | Myanmar | Asia |
| SRR12983342 | 1371 | 2017 | Myanmar | Asia |
| ERR298340 | 682 | 1997 | Australia | Australasia |
| ERR298341 | 483 | 2005 | Australia | Australasia |
| ERR298342 | 268 | 1997 | Australia | Australasia |
| ERR298343 | 849 | 2010 | Australia | Australasia |
| ERR298344 | 998 | 2011 | Australia | Australasia |
| ERR298346 | 999 | 2011 | Australia | Australasia |
| ERR298356 | 291 | 2002 | New_Caledonia | Australasia |
| ERR298358 | 292 | 2004 | New_Caledonia | Australasia |
| ERR298773 | 57 | 1990 | Philippines | Asia |
| ERR298774 | 98 | 1990 | Philippines | Asia |
| ERR298775 | 57 | 1990 | Philippines | Asia |
| ERR298776 | 63 | 1990 | Philippines | Asia |
| ERR298777 | unknown | 2011 | Philippines | Asia |
| ERR311036 | 246 | 1978 | Papua New Guinea | Australasia |
| ERR311040 | 149 | 1992 | Australia | Australasia |
| ERR311041 | 274 | 1992 | Papua New Guinea | Australasia |
| ERR311042 | 280 | 1992 | Fiji | Australasia |
| ERR311043 | 238 | 1993 | Australia | Australasia |
| ERR311045 | 1070 | 1994 | Australia | Australasia |
| ERR311046 | 125 | 1994 | Australia | Australasia |
| ERR311049 | 149 | 1995 | Australia | Australasia |
| ERR311050 | 126 | 1996 | Australia | Australasia |
| ERR311052 | 285 | 1996 | Australia | Australasia |
| ERR311054 | 126 | 1997 | Northern Territory, Australia | Australasia |
| ERR311057 | 130 | 1998 | Australia | Australasia |
| ERR311058 | 245 | 1998 | Australia | Australasia |
| ERR311059 | 278 | 1998 | Australia | Australasia |
| ERR311061 | 103 | 1999 | Australia | Australasia |
| ERR311062 | 112 | 1999 | Australia | Australasia |
| ERR539740 | 262 | 1997 | Australia | Australasia |
| ERR539745 | 239 | 2003 | Australia | Australasia |
| ERR539751 | 332 | 2004 | Australia | Australasia |
| ERR539755 | 442 | 2004 | Australia | Australasia |
| ERR539756 | 1075 | 2005 | Australia | Australasia |
| ERR539763 | 280 | 2008 | Fiji | Australasia |
