# Supplementary material for: Melioidosis Queensland: An analysis of clinical outcomes and genomic factors
Source: PLoS Negl Trop Dis. 2023 Oct 12;17(10):e0011697. doi: 10.1371/journal.pntd.0011697 (PMC10610085; doi:10.1371/journal.pntd.0011697)
Supplement: S3 Table — B. Bivariate and multivariate analysis of factors associated with mortality. (DOCX) [file pntd.0011697.s003.docx]

**S3 A Table. Bivariate and multivariate analysis of factors associated with bacteraemia**

| **Variable** | **Bacteraemia** | | | |
| --- | --- | --- | --- | --- |
|  | **Bivariate** | | **Multivariate** | |
|  | *p-value* | OR (95%CI) | *p-value* | OR (95%CI) |
| Age groups, years <50 | *0.4* | 1.3 (0.7-2.3) | *0.5* | 1.2 (0.6-2.3) |
| 50-69 | - | Ref | - | Ref |
| ≥70 | *0.03* | 2.2 (1.1-4.5) | *0.04* | 2.2 (1.0-4.5) |
| Male | *0.03* | 1.8 (1.1-3.1) | *0.03* | 1.9 (1.1-3.3) |
| Pneumonia | *<0.001* | 2.8 (1.6–4.8) | *<0.001* | 2.8 (1.6–4.9) |
| LPSA | *0.04* | 0.5 (0.2–0.9) | *0.1* | 0.6 (0.3–1.2) |

**S3 B Table. Bivariate and multivariate analysis of factors associated with mortality**

| **Variable** | **Dead** | | | |
| --- | --- | --- | --- | --- |
|  | **Bivariate** | | **Multivariate** | |
|  | *p-value* | OR (95%CI) | *p-value* | OR (95%CI) |
| Age groups, years <50 | *0.8* | 0.9 (0.5–1.8) | *0.7* | 1.2 (0.6–2.4) |
| 50-69 | - | Ref | - | Ref |
| ≥70 | *0.07* | 1.8 (0.9-3.6) | *0.2* | 1.6 (0.8-3.4) |
| Bacteraemia | *<0.001* | 5.4 (2.2-13.1) | *0.002* | 4.3 (1.7-10.5) |
| Pneumonia | *0.01* | 2.3 (1.2-4.4) | *0.06* | 2.0 (0.9-3.9) |
| YLF | *0.1* | 1.5 (0.9–2.6) | *0.3* | 1.4 (0.7–2.6) |
