# Supplementary material for: Melioidosis Queensland: An analysis of clinical outcomes and genomic factors
Source: PLoS Negl Trop Dis. 2023 Oct 12;17(10):e0011697. doi: 10.1371/journal.pntd.0011697 (PMC10610085; doi:10.1371/journal.pntd.0011697)
Supplement: S4 Table — (DOCX) [file pntd.0011697.s004.docx]

**S4 Table. Incidence of melioidosis by Health Service**

| **Health Service** | **Incidence per 100,000** |
| --- | --- |
| Cairns and Hinterland | No data |
| Central Queensland | 0.07 |
| Central West | 0.40 |
| Darling Downs | 0.02 |
| Gold Coast | 0.00 |
| Mackay | 1.09 |
| Metro North | 0.01 |
| Metro South | 0.00 |
| North West | 9.15 |
| South West | 0.19 |
| Sunshine Coast | 0.00 |
| Torres and Cape | No data |
| Townsville | 3.91 |
| West Moreton | 0.00 |
| Wide Bay | 0.02 |

The health services were divided into quartiles based on incidence, i.e. each band had the same number of health services. Only health services with incidence were considered in the quartile classification.
