# Supplementary material for: Melioidosis Queensland: An analysis of clinical outcomes and genomic factors
Source: PLoS Negl Trop Dis. 2023 Oct 12;17(10):e0011697. doi: 10.1371/journal.pntd.0011697 (PMC10610085; doi:10.1371/journal.pntd.0011697)
Supplement: S5 Table — (DOCX) [file pntd.0011697.s005.docx]

**S5 Table. Comparison of** **demographic, clinical, and virulence factors in relation to bacteraemia**

|  | **Non-bacteraemia** | **Bacteraemia** | ***p-value*** |
| --- | --- | --- | --- |
|  | N=82 | N=197 |  |
|  |  |  |  |
| **Age, median (IQR)** | 57 (41-63) | 59 (47-70) | *0.04* |
| **Age groups** |  |  |  |
| 18-49 | 26 (30%) | 62 (71%) | *0.1* |
| 50-69 | 43 (35%) | 81 (65%) |  |
| ≥70 | 13 (19%) | 54 (81%) |  |
|  |  |  |  |
| **Age >50** | 56 (29%) | 135 (71%) | *0.9* |
|  |  |  |  |
| **First Nation** | 24 (28%) | 61 (72%) | *0.8* |
|  | 58 (30%) | 136 (70%) |  |
| **Sex** |  |  |  |
| Female | 36 (38%) | 59 (62%) | *0.03* |
| Male | 46 (25%) | 138 (75%) |  |
| **Region** |  |  |  |
| Mackay | 2 (14%) | 12 (86%) | *0.1* |
| Bowen | 0 (0%) | 14 (100%) |  |
| Townsville | 48 (31%) | 106 (69%) |  |
| Mount Isa | 6 (38%) | 10 (62%) |  |
| Ingham | 5 (36%) | 9 (64%) |  |
| Mornington Island | 11 (34%) | 21 (66%) |  |
| **Diagnosis year** |  |  |  |
| 1996-2004 | 30 (28%) | 77 (72%) | *0.07* |
| 2005-2012 | 26 (41%) | 38 (59%) |  |
| 2013-2020 | 26 (24%) | 82 (76%) |  |
| **Pneumonia** |  |  |  |
| Yes | 40 (23%) | 138 (77%) | *<0.001* |
| No | 42 (45%) | 52 (55%) |  |
| **Novel-ST** |  |  |  |
| Yes | 43 (30%) | 100 (70%) | *0.8* |
| No | 39 (29%) | 97 (71%) |  |
| **LPSA** |  |  |  |
| Yes | 70 (32%) | 146 (68%) | *0.04* |
| No | 12 (19%) | 51 (81%) |  |
| ***fhaB*3** |  |  |  |
| Yes | 68 (30 %) | 158 (70%) | *0.6* |
| No | 14 (26%) | 39 (74%) |  |
| **YLF** |  |  |  |
| Yes | 40 (27%) | 109 (73%) | *0.3* |
| No | 42 (32%) | 88 (68%) |  |
| **BTFC** |  |  |  |
| Yes | 41 (33%) | 85 (67%) | *0.3* |
| No | 41 (27%) | 112 (73%) |  |
| ***bimA*_Bm_** |  |  |  |
| Yes | 13 (26%) | 37 (74%) | *0.6* |
| No | 69 (30%) | 160 (70%) |  |
