# Supplementary material for: Melioidosis Queensland: An analysis of clinical outcomes and genomic factors
Source: PLoS Negl Trop Dis. 2023 Oct 12;17(10):e0011697. doi: 10.1371/journal.pntd.0011697 (PMC10610085; doi:10.1371/journal.pntd.0011697)
Supplement: S6 Table — (DOCX) [file pntd.0011697.s006.docx]

**S6 Table. Bivariate associations with LPSA**

|  | **LPSA -** | **LPSA +** | ***p-value*** |
| --- | --- | --- | --- |
|  | N=66 | N=226 |  |
|  |  |  |  |
| **Age, median (IQR)** | 59.5 (46-72) | 57 (46-68) | *0.4* |
| **Age groups** |  |  |  |
| 18-49 | 22 (33%) | 71 (31%) | *0.6* |
| 50-69 | 26 (39%) | 104 (46%) |  |
| ≥70 | 18 (27%) | 51 (23%) |  |
|  |  |  |  |
| **Age >50** | 45 (68%) | 154 (68%) | *1* |
|  |  |  |  |
| **Sex, male** | 50 (76%) | 145 (64%) | *0.1* |
| **Region** |  |  |  |
| Mackay | 3 (5%) | 13 (7%) | *0.01* |
| Bowen | 4 (7%) | 10 (5%) |  |
| Townsville | 45 (78%) | 117 (59%) |  |
| Mount Isa | 0 (0%) | 17 (9%) |  |
| Ingham | 4 (7%) | 11 (5%) |  |
| Mornington Island | 2 (3%) | 31 (16%) |  |
| **Diagnosis year** |  |  |  |
| 1996-2004 | 23 (35%) | 93 (41%) | *0.3* |
| 2005-2012 | 13 (20%) | 55 (24%) |  |
| 2013-2020 | 30 (46%) | 78 (35%) |  |
|  |  |  |  |
| **Bacteraemia** | 51 (81%) | 146 (68%) | *0.04* |
| **Pneumonia** | 45 (71%) | 140 (64%) | *0.3* |
| **Novel-ST** | 37 (56%) | 113 (50%) | *0.4* |
| ***fhaB*3** | 33 (50%) | 204 (90%) | *<0.001* |
| **YLF** | 25 (38%) | 131 (58%) | *0.004* |
| **BTFC** | 41 (62%) | 91 (40%) | *0.002* |
| ***bimA*_Bm_** | 4 (6%) | 50 (22%) | *0.003* |
