# Supplementary material for: Melioidosis Queensland: An analysis of clinical outcomes and genomic factors
Source: PLoS Negl Trop Dis. 2023 Oct 12;17(10):e0011697. doi: 10.1371/journal.pntd.0011697 (PMC10610085; doi:10.1371/journal.pntd.0011697)
Supplement: S7 Table — (DOCX) [file pntd.0011697.s007.docx]

**S7 Table. Bivariate associations with fhaB3**

|  | **fhab3 -** | **fhab3 +** | **p-value** |
| --- | --- | --- | --- |
|  | N=55 | N=237 |  |
|  |  |  |  |
| **Age, median (IQR)** | 57 (46-73) | 58 (47-68) | *0.9* |
| **Age groups** |  |  |  |
| 18-49 | 21 (38%) | 72 (30%) | *0.3* |
| 50-69 | 19 (35%) | 111 (47%) |  |
| ≥70 | 15 (27%) | 54 (23%) |  |
|  |  |  |  |
| **Age >50** | 34 (62%) | 165 (70%) | *0.3* |
| **First Nation** | 19 (35%) | 68 (29%) | *0.4* |
| **Sex, male** | 41 (75%) | 154 (65%) | *0.2* |
| **Region** |  |  |  |
| Mackay | 4 (8%) | 12 (6%) | *0.04* |
| Bowen | 6 (12%) | 8 (4%) |  |
| Townsville | 33 (67%) | 129 (62%) |  |
| Mount Isa | 1 (2%) | 16 (8%) |  |
| Ingham | 3 (6%) | 12 (6%) |  |
| Mornington Island | 2 (4%) | 31 (15%) |  |
| **Diagnosis year** |  |  |  |
| 1996-2004 | 20 (36%) | 96 (41%) | *0.7* |
| 2005-2012 | 12 (22%) | 56 (24%) |  |
| 2013-2020 | 23 (42%) | 85 (36%) |  |
|  |  |  |  |
| **Bacteraemia** | 39 (74%) | 158 (70%) | *0.6* |
| **Pneumonia** | 43 (80%) | 142 (62%) | *0.01* |
| **Novel-ST** | 28 (51%) | 122 (52%) | *0.9* |
| **LPSA** | 22 (40%) | 204 (86%) | *<0.001* |
| **YLF** | 18 (33%) | 138 (58%) | *<0.001* |
| **BTFC** | 37 (67%) | 95 (40%) | *<0.001* |
| ***bimA*_Bm_** | 4 (7%) | 50 (21%) | *0.02* |
