# Supplementary material for: Melioidosis Queensland: An analysis of clinical outcomes and genomic factors
Source: PLoS Negl Trop Dis. 2023 Oct 12;17(10):e0011697. doi: 10.1371/journal.pntd.0011697 (PMC10610085; doi:10.1371/journal.pntd.0011697)
Supplement: S8 Table — (DOCX) [file pntd.0011697.s008.docx]

**S8 Table. Bivariate associations with YLF/BTFC**

|  | **YLF** | **BTFC** | ***p-value*** |
| --- | --- | --- | --- |
|  | N=156 | N=132 |  |
|  |  |  |  |
| **Age, median (IQR)** | 63 (53-73.5) | 50 (40-62) | *<0.001* |
| **Age groups** |  |  |  |
| **18-49** | 28 (18%) | 62 (47%) | *<0.001* |
| **50-69** | 76 (49%) | 53 (40%) |  |
| **≥70** | 52 (33%) | 17 (13%) |  |
|  |  |  |  |
| **Age >50** | 128 (82%) | 70 (53%) | *<0.001* |
| **First Nation** | 23 (15%) | 61 (46%) | *<0.001* |
| **Sex, male** | 100 (64%) | 94 (71%) | *0.2* |
| **Region** |  |  |  |
| Mackay | 11 (8%) | 5 (4%) | *<0.001* |
| Bowen | 7 (5%) | 7 (6%) |  |
| Townsville | 108 (78%) | 53 (46%) |  |
| Mount Isa | 3 (2%) | 14 (12%) |  |
| Ingham | 7 (5%) | 8 (7%) |  |
| Mornington Island | 3 (2%) | 28 (24%) |  |
| **Diagnosis year** |  |  |  |
| 1996-2004 | 56 (36%) | 58 (44%) | *0.2* |
| 2005-2012 | 34 (22%) | 32 (24%) |  |
| 2013-2020 | 66 (42%) | 42 (32%) |  |
|  |  |  |  |
| **Bacteraemia** | 109 (73%) | 85 (68%) | *0.3* |
| **Pneumonia** | 100 (65%) | 82 (65%) | *0.9* |
| **Novel-ST** | 62 (40%) | 84 (64%) | *<0.001* |
| ***fhaB*3** | 138 (89%) | 95 (72%) | *<0.001* |
| **LPSA** | 131 (84%) | 91 (69%) | *0.002* |
| ***bimA*_Bm_** | 12 (8%) | 39 (30%) | *<0.001* |
