# Supplementary material for: Melioidosis Queensland: An analysis of clinical outcomes and genomic factors
Source: PLoS Negl Trop Dis. 2023 Oct 12;17(10):e0011697. doi: 10.1371/journal.pntd.0011697 (PMC10610085; doi:10.1371/journal.pntd.0011697)
Supplement: S9 Table — (DOCX) [file pntd.0011697.s009.docx]

**S9 Table. Bivariate associations with bimA_Bm_**

|  | **bimA_Bm_ -** | **bimA_Bm_ +** | **p-value** |
| --- | --- | --- | --- |
|  | N=238 | N=54 |  |
|  |  |  |  |
| **Age, median (IQR)** | 58 (47-70) | 53.5 (40-64) | *0.05* |
| **Age groups** |  |  |  |
| **18-49** | 70 (29%) | 23 (43%) | *0.1* |
| **50-69** | 108 (45%) | 22 (41%) |  |
| **≥70** | 60 (25%) | 9 (17%) |  |
|  |  |  |  |
| **Age >50** | 168 (71%) | 31 (57%) | *0.06* |
| **First Nation** | 69 (29%) | 18 (33%) | *0.5* |
|  |  |  |  |
| **Sex, male** | 160 (67%) | 35 (65%) | *0.7* |
| **Region** |  |  |  |
| Mackay | 15 (7%) | 1 (2%) | *0.05* |
| Bowen | 7 (3%) | 7 (15%) |  |
| Townsville | 137 (65%) | 25 (54%) |  |
| Mount Isa | 14 (7%) | 3 (7%) |  |
| Ingham | 12 (6%) | 3 (7%) |  |
| Mornington Island | 26 (12%) | 7 (15%) |  |
| **Diagnosis year** |  |  |  |
| 1996-2004 | 93 (39%) | 23 (43%) | *0.3* |
| 2005-2012 | 52 (22%) | 16 (30%) |  |
| 2013-2020 | 93 (39%) | 15 (28%) |  |
|  |  |  |  |
| **Bacteraemia** | 160 (70%) | 37 (74%) | *0.6* |
| **Pneumonia** | 154 (66%) | 31 (61%) | *0.5* |
| **Novel-ST** | 105 (44%) | 45 (83%) | *<0.001* |
| ***fhaB*3** | 187 (79%) | 50 (93%) | *0.02* |
| **LPSA** | 176 (74%) | 50 (93%) | *0.003* |
| **YLF** | 144 (61%) | 12 (22%) | *<0.001* |
| **BTFC** | 93 (39%) | 39 (72%) | *<0.001* |
