# Supplementary material for: Melioidosis Queensland: An analysis of clinical outcomes and genomic factors
Source: PLoS Negl Trop Dis. 2023 Oct 12;17(10):e0011697. doi: 10.1371/journal.pntd.0011697 (PMC10610085; doi:10.1371/journal.pntd.0011697)
Supplement: Attachment — Submitted filename: reviewer comments_PNTD-23-00850.docx [file pntd.0011697.s011.docx]

The manuscript by Gassiep et al., describes the genomic characteristics of clinical *B. pseudomallei* isolates and clinical outcomes among patients diagnosed with melioidosis from different regions in Queensland. Some of the findings presented in this manuscript add more insights regarding the epidemiology of melioidosis. However, there are few aspects that the authors might consider elaborating a bit more in detail.

- For readers from outside Australia, it would be informative if the authors could provide a brief description of the baseline demographic data. Also, it would be interesting to see what proportion of population in these areas are involved in agriculture and what kind of crops/soil types are in these regions.
- Have there been any culture confirmed cases of melioidosis among pediatric population.
- The authors have reported the MLST of the isolates in this study. It would be interesting to see how these strains are related to the ST of B. pseudomallei isolates reported from elsewhere (both within and outside Australia). Further, it would be more interesting if the authors could report the genetic diversity of the study isolates from different regions using phylogenetic trees.
