# Supplementary material for: Melioidosis Queensland: An analysis of clinical outcomes and genomic factors
Source: PLoS Negl Trop Dis. 2023 Oct 12;17(10):e0011697. doi: 10.1371/journal.pntd.0011697 (PMC10610085; doi:10.1371/journal.pntd.0011697)
Supplement: Attachment — Submitted filename: Reviewer comments_PNTD-23-00850_All.docx [file pntd.0011697.s012.docx]

Response to reviewer comments:

**Reviewer #1: The methods were clear.**

**It was not clear what proportion of eligible cases were included ie. had available isolates for sequencing.**

- Thank you for your comment. Isolates for all patients included in this study were sequenced. Line 126: “The bacterial isolates from all 292 patients included in this study were recovered…”

**Reviewer #1: Table 2: Were first nations individuals over-represented with respect to their proportion of the local population?**

- This is correct thank you. Additional information regarding the population has been included: Line 102 “Queensland is the North Eastern state of Australia. As of 2021 the total population was approximately 5.2 million residents. First Nations people accounted for 4.6% of the population.” And also included in the discussion “There was a stark contrast between the different regions and the proportion of First Nation patients. Almost all patients from Mornington Island identified as FN compared with no FN patients in Bowen and only 1 in Mackay. Generally, this coincides with the specific demographics of each region, with 80% of Mornington Island residents identifying as FN (15). However, it is unclear why Bowen with an FN population similar to both Ingham and Townsville had no cases in that population.”

**Line 197: Was there any temporospatial clustering of ST identified? Similarly, how closely are the included ST related in the context of broader B. pseudomallei phylogeny.**

- Thank you for this comment. A figure including a phylogenetic tree and associated region and ST data has been included.

  **Line 205: Was there any co-association of genomic virulence factors? Was there any temporospatial clustering?**
- Thank you for these questions. There was no co-association of genomic virulence factors. A multivariate analysis was performed and did not demonstrate co-association. The results of the multivariate analysis are included in Supplementary Table 2 A & B.
- Temporospatial clustering was found. This has been included in Figure 2.

**214: BTFC in First nations – was this related to spatial distribution. Were FN populations over-represented with respect to the populations in the regions in which BTFC isolates were recovered.**

- Yes, there does appear to be an over-representation of FN populations in certain regions. This has been included in Table 3. Additionally, the BTFC does appear to coincide with these populations. This has been included in Supplementary Table 7.

**223: Did the single CAZ resistant isolate have a Hx of prior exposure? Similarly for the single SXT resistant isolate?**

- This is a very good question. Unfortunately, due to limited clinical data, we are unable to determine prior antibiotic exposure. This has been included in the limitations section: “Patient treatment and prior antibiotic exposure data were also not available.”

**Line 239 – Please provide interpretation for the MICs**

- Thank you for this comment. Interpretations have been included: “Notably, the phenotypic MICs were CAZ = 1mg/L (susceptible increased exposure) and MEM = 0.75 mg/L (susceptible).”

**Reviewer #1: Line 256; What were the incidence rates for TSV vs. NW (too broad range in the figure)**

Thank you for your question. The incidence for Townsville HHS was 3.9 per 100,000 residents and for North West HHS was 9.1 per 100,000 residents. This has been included in Supplementary Table 3.

**Line 268; Has there been a decline in melioid RFs among local FN populations? Is that what this line is suggesting?**

- Thank you for this comment. The sentence has been modified for clarification: “It is also important to note that clinical risk factor data such as prevalence of diabetes mellitus or immunosuppression are missing; further limiting comparison of these regions.”

**Reviewer #1: Figure 1. How were the bands determined? Given the wide range in incidence rates it might be clearer to list them.**

Thank you for your question. The authors agree with statement and have included additional information within Supplementary data.

**Supplementary Table 3. Incidence of melioidosis by Health Service**

| **Health Service** | **Incidence per 100,000** |
| --- | --- |
| Cairns and Hinterland | No data |
| Central Queensland | 0.07 |
| Central West | 0.40 |
| Darling Downs | 0.02 |
| Gold Coast | 0.00 |
| Mackay | 1.09 |
| Metro North | 0.01 |
| Metro South | 0.00 |
| North West | 9.15 |
| South West | 0.19 |
| Sunshine Coast | 0.00 |
| Torres and Cape | No data |
| Townsville | 3.91 |
| West Moreton | 0.00 |
| Wide Bay | 0.02 |

The health services were divided into quartiles based on incidence, i.e. each band had the same number of health services. Only health services with incidence were considered in the quartile classification.

**Figure 1. Is there incidence data available for Cairns and Hinterland, & Torres and Cape to help provide context?**

- Unfortunately, the authors do not have access to the Cairns and Hinterland & Torres and Cape data.

**Reviewer #1: This is an interesting and well written manuscript.

My main comment would be around the need to provide some broader context regarding the genomic diversity and how this fits into what is already known about the genomic diversity in Australia and internationally.  Similarly ST typing of a highly genetically diverse organism like B. pseudomallei may not capture the relatedness of isolates. It would have been interesting to see a phylogeny with isolates from this study placed into a broader national/international context.**

Thank you for this comment. An additional figure has been included in the manuscript: “Figure 2 A. Maximum likelihood phylogeny of 292 clinical *Burkholderia pseudomallei* isolates from Queensland with a global set of genomes (n = 174) rooted to the most ancestral *B. pseudomallei* strain (MSHR0668) Figure 2 B. Maximum likelihood phylogeny of the Queensland isolates rooted to *B. pseudomallei* K96243”

**Reviewer #2: The study population and area needs more description**

- Thank you for this comment. An additional paragraph has been included within the introduction: Queensland is the North Eastern state of Australia. As of 2021 the total population was approximately 5.2 million residents. First Nations people accounted for 4.6% of the population. The median age was 38 years and a male to female ratio of 0.97. Employment in Agriculture, Forestry, and Fishing accounted for 2.7% of the population, with men representing two-thirds of these employees (14). The primary land use industries in Queensland were diverse including but not limited to cattle, sugar cane, and sorghum (15).”

**Reviewer #2: results can be presented in more detail using phylogenetic trees**

- Thank you for this comment. The authors agree that a phylogenetic tree would be useful and has been included. Please see response to reviewer 1.

**Reviewer #3:**

**The manuscript by Gassiep et al., describes the genomic characteristics of clinical *B. pseudomallei* isolates and clinical outcomes among patients diagnosed with melioidosis from different regions in Queensland. Some of the findings presented in this manuscript add more insights regarding the epidemiology of melioidosis. However, there are few aspects that the authors might consider elaborating a bit more in detail.**

**For readers from outside Australia, it would be informative if the authors could provide a brief description of the baseline demographic data. Also, it would be interesting to see what proportion of population in these areas are involved in agriculture and what kind of crops/soil types are in these regions.**

- See first response to Reviewer 2.

**Have there been any culture confirmed cases of melioidosis among pediatric population.**

- Yes, 11 cases occurred in the pediatric population. This information has been included in Line 160: “Eleven cases occurred in paediatric patients (age <18).”

**The authors have reported the MLST of the isolates in this study. It would be interesting to see how these strains are related to the ST of B. pseudomallei isolates reported from elsewhere (both within and outside Australia). Further, it would be more interesting if the authors could report the genetic diversity of the study isolates from different regions using phylogenetic trees.**

- Thank you for this comment. A phylogenetic tree has been included. Please see response to reviewer 1.
